# Cervical Disc Arthroplasty for Isolated One-Level Degenerative Spine Disease: A Comprehensive Review of the Current Trends

**DOI:** 10.7759/cureus.90097

**Published:** 2025-08-14

**Authors:** Konstantinos Zygogiannis, Petros Christoforos Christakakis, Pavlos Gerasimidis, Vaia Grigoriou, Emmanouil Zervos, Konstantinos Manolakos, Dimitrios Koulalis

**Affiliations:** 1 Medicine, National and Kapodistrian University of Athens School of Medicine, Athens, GRC; 2 Orthopaedics, Evangelismos General Hospital, Athens, GRC; 3 Orthopaedics and Traumatology, Attikon University Hospital, Athens, GRC; 4 Sports Medicine, Orthopaedic Clinic, Athens Medical Center, Athens, GRC; 5 Emergency, Laiko General Hospital, Athens, GRC

**Keywords:** cervical myelopathy, cervical spine surgery, current practising trends, disc arthroplasty, one-level disc cervical pathology

## Abstract

Cervical myelopathy is a debilitating degenerative condition caused by spinal cord compression, which may affect multiple levels of the cervical spinal cord. It frequently necessitates surgical intervention when conservative treatment fails. While anterior cervical discectomy and fusion (ACDF) has long been the standard procedure, cervical disc arthroplasty (CDA) has emerged as a viable alternative, particularly for patients with isolated one-level disease. This comprehensive review, according to Preferred Reporting Items for Systematic Reviews and Meta-Analyses (PRISMA) guidelines, synthesizes current literature evaluating the safety, efficacy, and biomechanical advantages of CDA in treating isolated one-level cervical myelopathy and/or radiculopathy. Evidence from current scientific papers demonstrates that CDA provides comparable neurological improvement to ACDF, since the process of decompressing is the same for both of them, while offering superior motion preservation, reduced rates of adjacent segment degeneration (ASD), and lower reoperation rates. Patient selection remains critical, with radiological and clinical criteria guiding candidacy for CDA. Technological advances, including 3D-printed implants and robotic-assisted surgery, are further enhancing outcomes and expanding indications. As long-term data accumulate continuously, CDA is increasingly supported as an effective, motion-preserving option for appropriately selected patients with single-level cervical myelopathy.

## Introduction and background

Cervical myelopathy is a significant neurological condition predominantly caused by compression of the cervical spinal cord, often resulting from degenerative changes associated with age, such as cervical spondylosis and disc herniation [1]. The clinical presentation of cervical myelopathy can vary widely, including symptoms like neck pain, weakness in the arms and hands, sensory changes, and balance difficulties. In its advanced stages, patients may experience severe motor dysfunction and debilitating impacts on daily activities [2]. The presence of classic MRI findings in conjunction with clinical symptoms is paramount for accurate diagnosis and necessitates vigilant assessment before considering surgical intervention and remains the gold standard for the diagnosis; however, distinct approaches like dynamic MRI assist in diagnosing this condition, revealing critical dynamic changes not evident in static imaging [3,4].

Surgical intervention is indicated primarily when conservative management fails to alleviate symptoms in the case of a radiculopathy or in cases of myelopathy to prevent neurologic deterioration. The clinical consensus is that decompression surgery should be considered in cases where significant cord compression is coupled with myelopathic symptoms [5]. This becomes particularly critical in older adults, where cervical spondylotic myelopathy remains a leading cause of spinal cord-related dysfunction [6]. As surgical techniques evolve, the decision-making process involves weighing the urgency of surgical intervention against the individual risk-benefit profile, with guidelines suggesting early intervention to preserve cognitive and physical function [7, 8].

The evolution of surgical techniques in managing cervical myelopathy reflects broader strides in spinal surgery, moving from traditional anterior cervical discectomy and fusion (ACDF) to cervical disc arthroplasty (CDA). ACDF has long been the gold standard for addressing cervical disc disease, offering effective decompression of the spinal cord and stabilization of the affected segment [9]. However, CDA has garnered attention for its purported biomechanical advantages, including better preservation of motion and reduced risk of adjacent segment degeneration (ASD) due to less rigid stabilization [10]. Studies highlight the statistical superiority of CDA over ACDF in terms of neurological recovery and quality of life outcomes [11], suggesting that its use is increasingly favored in appropriately selected patients with degenerative cervical myelopathy [12]. Therefore, as clinical evidence continues to support its efficacy, CDA may soon represent the preferred method for treating specific subsets of patients with cervical myelopathy under specific indications.

## Review

Materials and methods

**Figure 1:**
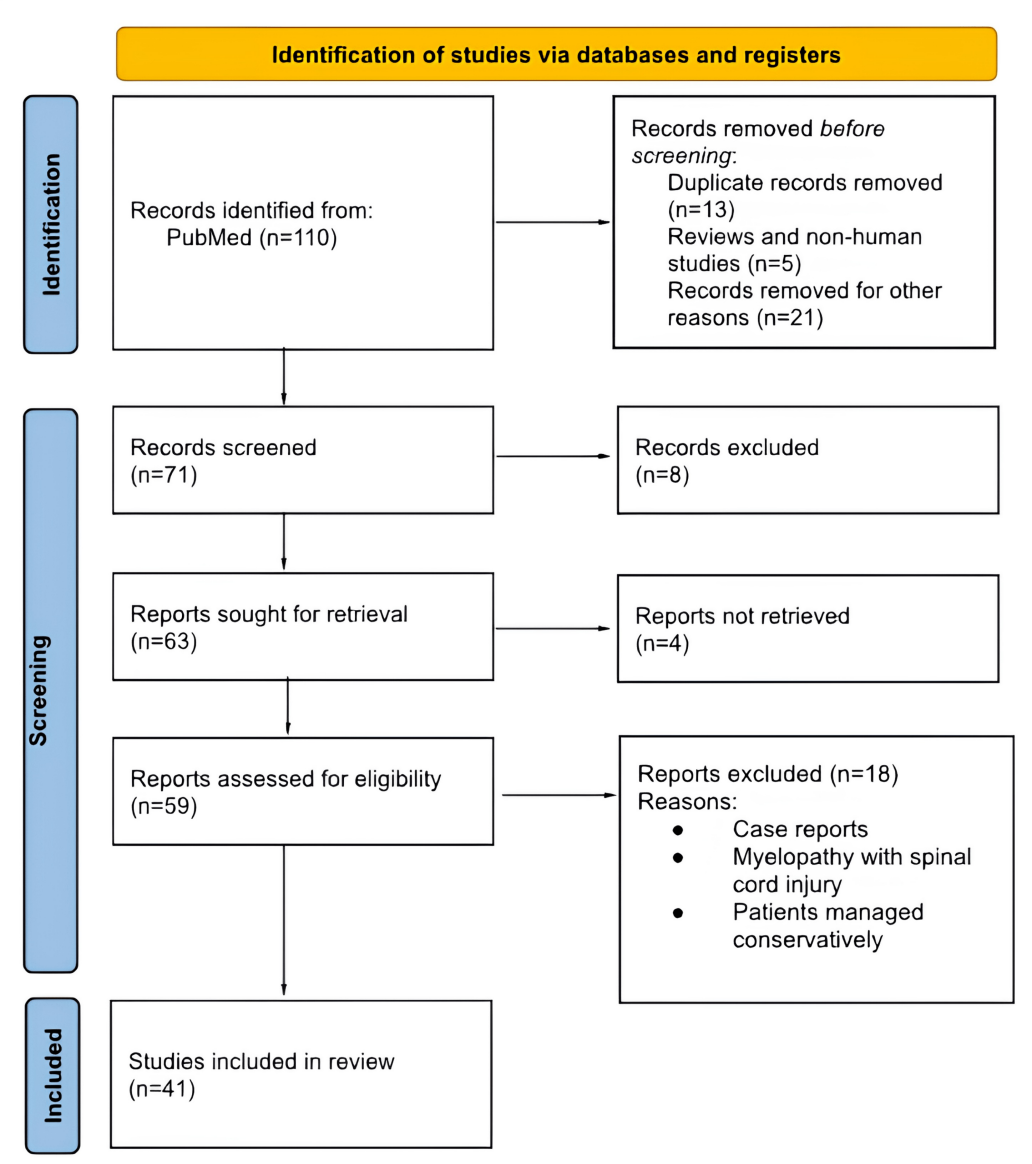
Flowchart of the review study according to PRISMA guidelines PRISMA: Preferred Reporting Items for Systematic Reviews and Meta-Analyses

A comprehensive literature review was conducted following the Preferred Reporting Items for Systematic Reviews and Meta-Analyses (PRISMA) guidelines to identify studies comparing ACDF with CDA for the treatment of one-level degenerative cervical spine disease. Eligible studies included original peer-reviewed articles involving human subjects that specifically examined surgical outcomes of ACDF versus CDA with symptoms of radiculopathy and/or myelopathy. Studies were excluded if they focused solely on conservative treatment, involved patients with acute spinal cord injury, addressed multi-level cervical disease, or were case reports without a literature review. No exclusions were made based on the type or brand of prosthesis in order to avoid potential conflicts of interest. The literature search was performed across multiple electronic databases, including PubMed, Web of Science, Cochrane Library, Scopus, and Excerpta Medica database (EMBASE), using combinations of MeSH terms and keywords such as “cervical spine myelopathy,” “one-level degenerative cervical spine disease,” “anterior cervical discectomy and fusion,” “cervical disc arthroplasty,” and “treatment for one-level surgical pathology.” Additional manual searches were performed in the archives of key specialty journals, including Spine, European Spine Journal, and Journal of Bone and Joint Surgery. Two reviewers independently screened titles and abstracts to identify eligible studies, with full texts assessed for final inclusion based on predefined criteria. Data extraction was carried out independently by both reviewers using a standardized form, collecting information on study design, sample size, patient demographics, type of intervention, follow-up duration, clinical and radiological outcomes, complication rates, reoperations, and funding sources. Disagreements during study selection or data extraction were resolved through discussion or consultation with a third reviewer. Risk of bias was assessed using the Cochrane Risk of Bias Tool for randomized controlled trials and the Newcastle-Ottawa Scale for observational studies. Figure 1 presents a PRISMA flowchart outlining the study selection process.

Review

Patient Selection Criteria

The selection of patients for CDA due to isolated one-level cervical myelopathy involves careful consideration of both clinical and radiological criteria. Clinically, successful candidates typically present with moderate to severe myelopathic symptoms, including motor deficits and sensory changes, that can be directly linked to a singular level of cervical compression observable on imaging [13]. Radiologically, criteria for CDA candidacy include the presence of single-level degenerative disc disease confirmed through MRI or CT, which shows significant spinal cord compression and corresponding neurological deficits [14,15]. A neurophysiological evaluation may also support the diagnosis by revealing compromised spinal cord function attributed to the observed imaging findings [13].

Patient selection necessitates excluding candidates who exhibit specific criteria that may compromise surgical outcomes and overall health. Key exclusion factors include spinal instability, severe spondylotic changes, and conditions like ossification of the posterior longitudinal ligament (OPLL) [16]. Such factors can complicate the surgical procedure and may lead to unfavorable postoperative outcomes. Additionally, patients with multifactorial cervical myelopathy from multiple affected levels may not benefit from CDA, which is primarily aimed at addressing isolated compressive pathology [17]. Each patient's overall health status and comorbid conditions must be considered to ensure they are fit for elective surgery, which may also involve assessments of other concurrent health risks that could impact recovery and rehabilitation post surgery [18].

The role of preoperative imaging remains crucial in the assessment process for candidates undergoing one-level CDA for cervical myelopathy. MRI is seen as the gold standard for diagnosing and evaluating the extent of cervical compressive myelopathy, effectively visualizing anatomical structures and any significant cord signal changes [19]. Dynamic imaging techniques, including dynamic X-rays, can additionally provide insights into the stability of the cervical spine, identifying any segmental instability that may necessitate reconsideration of CDA suitability [20]. In cases where MRI findings indicate significant intramedullary high signal intensity, there is a potential correlation with worse clinical outcomes post-surgery, emphasizing the importance of comprehensive preoperative imaging for surgical planning [21]. Overall, a thorough radiological assessment combined with clinical evaluation allows for tailored decision-making regarding CDA in patients with isolated one-level cervical myelopathy.

Outcomes of CDA in One-Level Myelopathy

CDA has demonstrated substantial efficacy in treating patients with isolated one-level cervical myelopathy, yielding marked improvements in both neurological status and functional outcomes. Clinical studies consistently show that patients undergoing CDA experience significant postoperative gains, as evidenced by well-established measures such as the Neck Disability Index (NDI), the Visual Analog Scale (VAS) for neck and arm pain, and the Japanese Orthopaedic Association (JOA) score. For example, in a long-term follow-up study involving patients who underwent one-level CDA using the Prestige-LP device (Medtronic plc, Dublin, Ireland), mean VAS scores for neck pain decreased from 6.0 ± 2.2 to 2.0 ± 1.4, while arm pain decreased from 6.2 ± 2.5 to 1.9 ± 1.4 (p < 0.001 for both). NDI scores improved from 33.9 ± 10.1 preoperatively to 12.9 ± 5.4 postoperatively (p < 0.001), and JOA scores showed similar significant improvements, reflecting neurological recovery and enhanced functional capacity [22].

These improvements are not only significant in the early postoperative period but are also maintained long-term. For instance, in a recent study with a mean follow-up of 38 months, range of motion (ROM) was preserved in the operated segments, and the reoperation rate was as low as 3.2%, with minimal adjacent-level motion abnormalities [23]. These outcomes affirm the durability of neurological benefits and the biomechanical integrity offered by motion-preserving CDA implants [22,23].

Comparative data between CDA and ACDF reinforce the clinical value of CDA. In a retrospective cohort study involving 122 CDA and 108 ACDF patients, both groups experienced regression of intramedullary spinal cord signal on MRI postoperatively. However, only the CDA group showed significant improvement in VAS scores at 24 months, suggesting enhanced pain relief following arthroplasty [24]. Furthermore, a meta-analysis of eight randomized controlled trials with follow-up periods of four to seven years demonstrated that CDA resulted in a 45% reduction in overall secondary surgical procedures (relative risk (RR) = 0.55; 95% confidence interval (CI): 0.42-0.73; p < 0.0001) and a 60% reduction in index-level revisions (RR = 0.40; 95% CI: 0.28-0.58; p < 0.00001) when compared to ACDF. Neurological and NDI success rates were also significantly higher in the CDA cohorts [25,26].

One of the most compelling arguments for CDA is its ability to preserve segmental mobility, which has been associated with a lower incidence of ASD. A systematic review and meta-analysis published in the Spine journal analyzed 14 randomized trials involving 3,235 patients with follow-ups ranging from two to seven years. The study reported a 43% reduction in the risk of symptomatic ASD (RR = 0.57; 95% CI: 0.37-0.87; p = 0.009) and a 53% reduction in adjacent-segment reoperation rates (RR = 0.47; 95% CI: 0.32-0.70; p = 0.0002) in favor of CDA [22]. A more recent meta-analysis from 2024, evaluating 10-year outcomes, found that CDA was associated with improved NDI and VAS scores, a reduced need for secondary interventions, and fewer adverse events. Although the functional benefits were statistically significant, they did not reach the minimum clinically important difference in all cases. Nevertheless, neurological success rates were comparable to those of ACDF, further supporting CDA as an effective alternative for long-term management (Table 1) [26].

**Table 1:** Postoperative outcome scores of cervical disc arthroplasty versus anterior cervical discectomy and fusion Outcomes of CDA compared to ACDF demonstrate substantial efficacy in treating patients with isolated one-level cervical myelopathy, yielding marked improvements in both neurological status and functional outcomes. VAS: Visual Analog Scale; NDI: Neck Disability Index; JOA: Japanese Orthopedic Association; ROM: range of motion; ASD: adjacent segment disease; CDA: cervical disc arthroplasty; RR: relative risk; ACDF: anterior cervical discectomy and fusion

Measure	CDA (one‑level)	ACDF	Relative advantage
VAS: neck/arm [22]	Preop neck: 6.0, Postop: 2.0/Preop arm: 6.2, Postop: 1.9	Significant, but less pronounced; long-term trend favors CDA	CDA slightly better
NDI score [22]	Preop: 33.9 → Postop: 12.9	Comparable in the long term, CDA showed statistical but not clinical superiority	Comparable/slight CDA benefit
JOA score [22]	Significant improvement maintained	Similar neurological outcomes	Equivalent
ROM preservation [22,23,27]	Maintained ~9°, ~80% mobility at 6+ years	Minimal to no motion post-op	CDA major advantage
Secondary procedures [26]	RR 0.55 (45% lower) total; RR 0.40 (60% lower) index-level	Baseline	CDA advantage
Adverse events [26,27]	Fewer complications & secondary surgeries	Slightly higher	CDA preferable

Complementary findings from a 2020 meta-analysis in BMC Neurology, which included 13 randomized controlled trials with an average follow-up of nearly seven years, confirmed that CDA offers superior clinical success (odds ratio (OR) = 1.54; 95% CI: 1.15-2.08; p = 0.004) compared to ACDF. The study also reported significantly better ROM preservation (mean difference = 1.77°; 95% CI: 1.60-1.95; p < 0.001), lower ASD risk (OR = 0.51; 95% CI: 0.35-0.76; p = 0.001), and a 59% reduction in reoperation rates (OR = 0.41; 95% CI: 0.25-0.69; p = 0.001) in the CDA group [27].

Comparison Between CDA and ACDF

The comparative analysis between CDA and ACDF reveals significant differences in both radiological and clinical outcomes for cervical degenerative disease. A meta‑analysis encompassing 13 randomized controlled trials with a mean follow‑up of 83.1 months demonstrated that CDA provides improved overall clinical success (OR 1.54; 95% CI: 1.15-2.08; p = 0.004) and better preservation of ROM (mean difference 1.77°; 95% CI: 1.60-1.95; p < 0.001) compared to ACDF. Importantly, CDA significantly reduced the incidence of adjacent segment degeneration (ASD) (OR 0.51; 95% CI: 0.35-0.76; p = 0.001) and reoperation rates (OR 0.41; 95% CI: 0.25-0.69; p = 0.001) [27]. Additional evidence supports CDA’s long-term advantages. A 2024 meta-analysis of six studies with 10-year follow-up reported statistically significant but not clinically important improvements in NDI and VAS scores for CDA patients relative to ACDF (p < 0.05), alongside a markedly lower rate of secondary surgeries and adverse events (p < 0.05). Neurological success, measured by JOA scores, remained equivalent between the two groups [26].

Longitudinal data on adjacent-level operations are particularly revealing. In one comprehensive analysis of 11 randomized controlled trials, CDA and ACDF showed similar adjacent-level surgery rates at two years: 2.3% for CDA versus 3.6% for ACDF. However, over five to seven years, divergence became statistically significant: CDA exhibited a 4.3% rate versus 10.8% for ACDF (p < 0.001). Index-level reoperations were consistently fewer in the CDA group (5.2% vs. 12.7%; p < 0.001) [28]. Furthermore, recent pooled analysis indicates CDA offers a higher overall surgical success rate (OR 2.71; 95% CI: 1.95-3.77) and significantly reduced secondary surgery (OR 0.254; 95% CI: 0.169-0.382) and complication rates (OR 0.548; 95% CI: 0.326-0.919), compared to ACDF. Improvements in neck pain VAS were notably higher in CDA patients, although changes in arm pain VAS, NDI, and JOA scores did not differ significantly between procedures (Table 2) [29].

**Table 2:** Long-term comparison of postoperative outcomes regarding CDA vs. ACDF from the available studies ROM: range of motion; NDI: Neck Disability Index; VAS: Visual Analog Scale; OR: odds ratio; CI: confidence interval; CDA: cervical disc arthroplasty; ACDF: anterior cervical discectomy and fusion

Outcome measure	CDA	ACDF	Statistical significance
Clinical success (overall) [26-29]	OR 1.54 (95% CI: 1.15–2.08)	Baseline	p = 0.004 (researchgate.net, pubmed.ncbi.nlm.nih.gov)
ROM preservation [27]	+1.77° (95% CI: 1.60–1.95)	Baseline	p < 0.001
Adjacent segment disease [27]	OR 0.51 (95% CI: 0.35–0.76)	Baseline	p = 0.001
Reoperation (index and adjacent) [28]	OR 0.41 (95% CI: 0.25–0.69)	Baseline	p = 0.001
10-year NDI and VAS improvement [26]	Statistically improved	Compared to CDA	p < 0.05
Timepoint: adjacent-level surgery [27]	2 years: 2.3%, 7 years: 4.3%	2 years: 3.6%; 7 years: 10.8%	p < 0.05
Surgical success (two-level cases) [29]	OR 2.71 (95% CI: 1.95–3.77)	Baseline	–
Secondary surgery rate [29]	OR 0.254 (95% CI: 0.169–0.382)	Baseline	–
Complications [29]	OR 0.548 (95%% CI: 0.326–0.919	Baseline	–

Biomechanical and Kinematic Considerations

Biomechanical and kinematic considerations are critical when evaluating CDA versus ACDF, particularly regarding segmental ROM at the index level and its impact on adjacent segments. CDA is explicitly engineered to maintain normal cervical biomechanics, thereby significantly preserving segmental mobility (Table 3). A large meta-analysis of randomized controlled trials and cohort studies confirmed that CDA confers significantly better ROM preservation compared to ACDF (mean difference +1.77°; 95% CI: 1.60-1.95; p < 0.001) at long-term follow-up, with corresponding improvements in clinical outcomes and reduced incidence of ASD and reoperations [27,30]. Prospective clinical data comparing CDA and ACDF reinforce these biomechanical benefits. Meanwhile, a 2024 network meta-analysis evaluated constrained, semiconstrained, and unconstrained CDA devices against ACDF. It concluded that all CDA variants significantly reduced ASD risk (p ≤ 0.03), with semiconstrained and unconstrained devices also showing lower reoperation rates than both constrained CDA and ACDF. Unconstrained devices preserved significantly greater ROM than ACDF (p < 0.001) [31].

**Table 3:** Biomechanical and kinematic considerations regarding postoperative outcomes of CDA vs. ACDF CDA is explicitly engineered to maintain normal cervical biomechanics, thereby significantly preserving segmental mobility. CDA: cervical disc arthroplasty; ACDF: anterior cervical discectomy and fusion; ASD: adjacent segment disease; ROM: range of motion; RR: relative risk

Outcome measure	CDA results	ACDF results	Significance and source
Index-level ROM preservation [27,30]	↑ +1.77° (95% CI: 1.60–1.95); p	Minimal to no postoperative ROM	Highly significant difference
Risk of adjacent segment degeneration [27,30]	RR significantly reduced; p ≤ 0.03 across device types	Baseline	Statistically lower ASD risk with CDA
Global cervical ROM (C2–C7) [31]	Maintained/increased postoperatively	Decreased from ~39.2° to ~32.2°; p	Highly significant difference
Segmental vs. global stiffness [27,30,31]	Closer to physiological stiffness; lower postoperative stiffness	Higher stiffness compared to CDA	Biomechanical advantage supports better load distribution
Long-term alignment a load distribution [27,30,31]	Maintained over five to 10 years; fewer ASD and reoperations	Baseline	Long-term biomechanical stability and fewer complications

Kinematic studies further demonstrate that CDA maintains postoperative global and segmental stiffness closer to physiological levels, unlike ACDF, which often increases stiffness at adjacent levels. Meta-analyses of clinical and biomechanical studies have consistently shown CDA patients to have lower rates of ASD and fewer reoperations compared to fusion groups [32]. Prosthesis design and surgical technique profoundly influence biomechanical and alignment outcomes. Unconstrained devices typically better mimic natural disc behavior, retaining superior ROM and reducing the need for index-level reoperation [31]. Implant material and position, coupled with careful surgical placement, are key to preserving postoperative alignment and load distribution. Long-term follow-up (up to 10 years) indicates that properly implanted CDA prostheses maintain cervical alignment and motion, thereby reducing the risk of degeneration and subsequent surgeries at both the index and adjacent levels [27, 30-32].

Current Trends and Technological Advances

Current trends and technological advancements in CDA reflect the growing acceptance and the evolving landscape of surgical techniques in the management of degenerative cervical disc disease. The uptake of CDA in clinical practice worldwide has shown a significant upward trajectory, particularly in the United States, where it has experienced an annual growth rate of approximately 20% as a primary intervention and around 6% for revision surgeries [33]. This trend is driven by the favorable outcomes associated with CDA, including improved patient-reported outcomes compared to ACDF, which has traditionally been considered the gold standard treatment [34]. Increased awareness of the benefits associated with motion preservation and reduced rates of ASD has enhanced provider and patient confidence in CDA as a treatment option.

The evolution of surgical training, guidelines, and insurance approvals also plays a critical role in the dissemination of CDA. Following FDA approval of the initial CDA devices, the development of comprehensive training programs aimed at teaching the nuances of this technique has been instrumental in facilitating its adoption [35]. Regulatory bodies and professional organizations have issued guidelines that outline the indications for CDA, creating a clearer pathway for insurance coverage aligned with evolving evidence on clinical effectiveness. These shifts are crucial in broadening access to CDA, as clinicians are increasingly supported by evidence demonstrating its efficacy over ACDF in specific patient populations [36,37].

Innovations in CDA technology have further propelled its adoption and refinement. Notable advancements include the development of 3D-printed discs and customized implants tailored to the unique anatomical needs of individual patients. These innovations enable improved fit and functionality, thus enhancing surgical outcomes [38]. Additionally, the incorporation of robotic assistance and navigation systems in the surgical process offers improved accuracy and reproducibility during implantation, which can lead to better postoperative alignment and function [10, 39]. These technological developments are enhancing the performance of CDA and are setting the stage for further exploration of hybrid surgical techniques and improved patient outcomes in cervical spine surgery [40, 41].

Limitations

This review on CDA for isolated one-level myelopathy is subject to several limitations. A major concern is the heterogeneity among included studies, with variations in patient selection, surgical techniques, prosthesis types, and outcome measures, which hampers direct comparison and limits generalizability. Often, the sole goal of surgical decompression is to stabilize the patient in his current condition and not improve due to the nature of the disease; thus, the satisfaction and outcome scores may differ greatly. Additionally, literature specifically addressing isolated myelopathy as opposed to radiculopathy or mixed pathology is relatively scarce, resulting in potentially limited evidence base. The lack of long-term follow-up data further restricts conclusions about implant durability and adjacent segment disease. Most available studies are retrospective or observational in nature, with few randomized controlled trials, thereby reducing the overall strength of evidence. Moreover, potential publication bias and inconsistent diagnostic criteria for myelopathy across studies may further skew findings. Finally, the rapidly evolving landscape of CDA technology and surgical practice may render some included data outdated, limiting the applicability of the review to current clinical settings.

## Conclusions

In conclusion, CDA can prove highly effective for an isolated one-level cervical spine disease, as neck pain VAS, NDI, and JOA scores show promising results from various studies. When compared with ACDF, NDI scores can be positive for both of them; clinically, there is no significant difference. Since CDA is a motion-preserving surgical technique, while with ACDF there is fusion of the respective spine level, the rate of adjacent segment disease is higher in the ACDF group. Finally, even though there are statistical differences between those two groups concerning the neurologic recovery and radiculopathy, we consider them patient-specific and not of clinical importance since the process of surgical approach and decompression is the same for both types of operation, and the quality of it can vary depending on the surgeon's experience and the patient's individualized factors.
